# Bryonolic Acid, a Triterpenoid, Protect Against *N*-methyl-d-Aspartate-Induced Neurotoxicity in PC12 Cells

**DOI:** 10.3390/molecules21040418

**Published:** 2016-03-28

**Authors:** Jinhua Que, Miao Ye, Yuqin Zhang, Wen Xu, Huang Li, Wei Xu, Kedan Chu

**Affiliations:** College of Pharmacy, Fujian University of Traditional Chinese Medicine, Minhou Shangjie, Fuzhou 350122, Fujian, China; qjhfjtcm@sina.com (J.Q.); AS2006041098@163.com (M.Y.); zyqfj@hotmail.com (Y.Z.); xwfjtcm@126.com (W.X.); lihuang3413@gmail.com (H.L.); xwfjlab@163.com (W.X.)

**Keywords:** bryonolic acid, PC12 cells, Ca^2+^, Bcl-2, Bax, p-CaMKII, p-CREB

## Abstract

Calcium overload is considered to be one of the mechanisms of cerebral ischemia. Ca^2+^ influx and Ca^2+^/calmodulin-dependent protein kinase II (CaMKII) and cAMP response element-binding protein (CREB) phosphorylation are considered to be involved in *N*-Methyl-d-aspartate (NMDA)-induced apoptosis process. This study investigated the neuroprotective effects of bryonolic acid (BA) in an NMDA-induced rat adrenal pheochromocytoma cell line (PC12) cells and the potential mechanism. PC12 was treated by NMDA to establish an excitotoxicity model. BA (110,100 and 1000 μM final concentration) was added to the medium 24 h prior to the addition of NMDA. Subsequently, a methyl thiazolyl tetrazolium (MTT) assay and a lactate dehydrogenase (LDH) release were performed. Ca^2+^ concentration was demonstrated using a scanning-dual wavelength fluorimetric method. In addition, protein and mRNA levels were determined via Western blot and real-time PCR. In the presence of BA, MTT assay and LDH assay showed that more cells were viable in comparison with the NMDA group. Moreover, the concentration of Ca^2+^ decreased with the addition of BA in culture. Furthermore, BA could upregulate protein expressions of Bcl-2, p-CREB, and p-CaMKII and downregulate protein expression of Bax. The mRNA results showed that the pattern of mRNA expression were similar to their respective protein levels. All these results indicate that BA protected PC12 cells against NMDA-induced apoptosis by inhibiting Ca^2+^ influx and regulating gene expression in the Ca^2+^-CaMKII-CREB signal pathway. Therefore, the present study supports the notion that BA may be a promising neuroprotective agent for the treatment of cerebral ischemia disease.

## 1. Introduction

Ischemic stroke is by far the most common kind of stroke that accounts for about 88% of all strokes. It is the major cause of disability and the third cause of death across the world [1]. Its occurrence still increases with advanced age according to the World Health Organization. The mechanisms involved in ischemic stroke is complex and not fully understood, and excitotoxicity, calcium overload, oxidative stress, inflammation, and apoptosis [2,3,4,5] are involved. It was proved that cerebral ischemic injury was related to massive influx of extracellular Ca^2+^ [6,7]. Calcium overload is regarded as the final common pathway of the action mechanisms leading to neuronal death [8,9,10]. Therefore, the regulation of Ca^2+^ concentration may play an important role in preventing pathological apoptosis in cerebral ischemia.

Intracellular Ca^2+^ concentration is at the level of about 10^−8^–10^−7^ mol/L under the stable condition of a cell. However, when the cell is stimulated, Ca^2+^ concentration is too high; when intracellular Ca^2+^ concentration is 10^−6^ mol/L or above, CaM is activated by combining with Ca^2+^. Then, it further triggers Ca^2+^ dependent cascade reactions. Ca^2+^ overload activates Ca^2+^/calmodulin-dependent protein kinase II (CaMKII), which is a serine/threonine protein kinase and is made up 1%–2% of the total protein in certain regions of the brain [11,12,13]. CaMKII is an important member of the calcium/calmodulin-activated protein kinase family, functioning in neural synaptic stimulation and T-cell receptor signaling [14,15]. CaMKII has catalytic and regulatory domains. The binding of Ca^2+^/calmodulin to its regulatory domain releases its autoinhibitory effect and activates the kinase [16]. When the Ca^2+^/calmodulin (CaM) complex combines with CaMKII, CaMKII is activated and further autophosphorylates at Thr286 to render the kinase constitutively active [16]. Phosphorylation of CaMKII is important in controlling a variety of cellular functions [17,18]. Previous reports demonstrated that the autophosphorylation of CaMKII might be an important process in the regulation of ischemia [19,20].

CaMKII as a protein kinase can activate a cAMP response element-binding (CREB) protein. The activated CREB protein then binds to a CRE region and is then bound to by a CBP (CREB-binding protein), which coactivates it, allowing it to switch certain genes on or off.

CREB is a transcription factor present in numerous tissues, it plays a large regulatory role in the nervous system [21,22]. CREB is believed to play a key role in promoting neuronal survival, precursor proliferation, neurite outgrowth, and neuronal differentiation in certain neuronal populations [23,24,25]. CREB is activated by phosphorylation at Ser133 via protein kinase C (PKC), CaMK, *etc.* [12] and is also able to selectively activate numerous downstream genes and its binding sequences have been identified in different genes, such as Bcl-2 [26]. Therefore, CaMKII and CREB are important pharmacological targets of intersection for different pathways involved in neuronal apoptosis.

**Figure 1:**
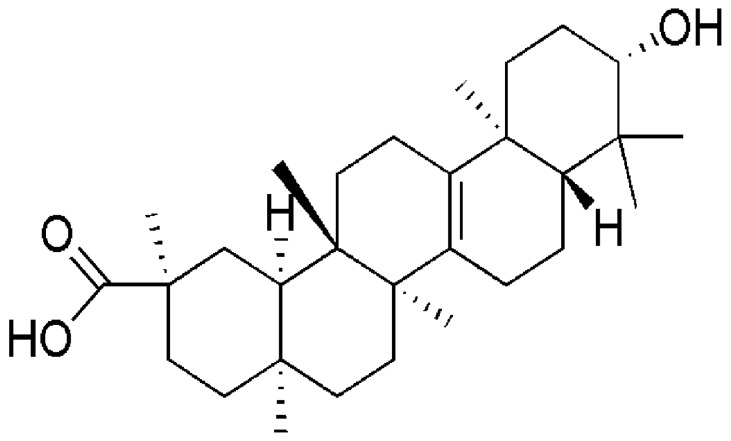
Chemical constitution of bryonolic acid (BA).

Trichosanthis radix is the dry radix of *Trichosanthes kirilowii* Maxim. or *Trichosanthes rosthornii* Herms (recorded in China Pharmacopoeia), which is often used to treat neurological symptoms in China. Some studies have demonstrated that trichosanthis radix has various pharmacological actions on cerebral ischemic injury, such as neuroprotective effects [27] and anti-apoptosi [28,29]. Bryonolic acid (3-hydroxy-D:C-friedoolean-8-en-29-oic acid) (BA), as shown in Figure 1, is a triterpenoid that was isolated from trichosanthis radix. Several biological activities have been reported for BA, including antiallergic properties [30], cytotoxic and antitumor activities in cancer cell lines [31,32,33], and anti-inflammatory and antioxidant properties [34]. Based on the aforementioned, we hypothesized that BA possibly exerts its neuroprotective effects on *N*-methyl-d-aspartate (NMDA)-induced neurotoxicity and modulation of the CaMKII signaling pathway. Herein, present studies were carried out to investigate whether BA protects against NMDA-induced neurotoxicity via modulation of the CaMKII signaling pathway in PC12 cells.

## 2. Results

### 2.1. Effect of BA on the Cell Viability in NMDA-Induced Excitotoxicity

**Figure 2:**
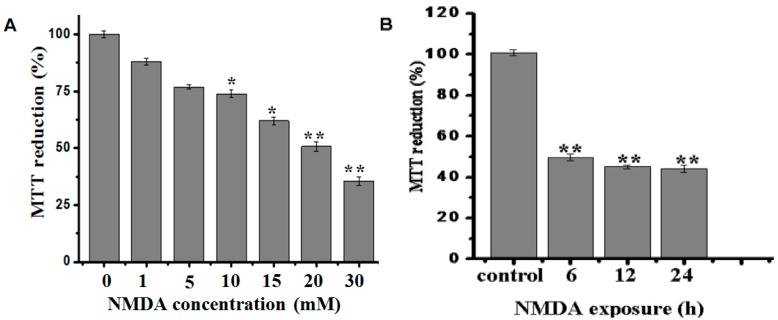
PC12 cells were treated with NMDA at the indicated concentrations for 12 h or with 20 mM NMDA for indicated time (as determined by MTT assay). (**A**) PC12 cells were treated with 1, 5, 10, 15, 20, 30 mM NMDA for 12 h; (**B**) PC12 cells were treated with 20 mM NMDA for 6, 12, 24 h. Data are expressed as mean ± SD; *n* = 8 wells for each group; * *p* < 0.05, ** *p* < 0.01, compared to control (one-way ANOVA).

Firstly, the dose response and time course of NMDA-induced cell excitotoxicity were determined. PC12 cells (2 × 10^4^ cells per well) were exposed to various concentrations of NMDA (1–30 mM) for 12 h or to 20 mM NMDA for various time periods (6, 12, and 24 h). The results showed that the cell viability was inhibited by NMDA in a concentration-dependent manner and a time-dependent manner (Figure 2A,B). Thus, cell injury induced 6 h exposure to NMDA at 20 mM, which was chosen for the subsequent experiments.

**Figure 3:**
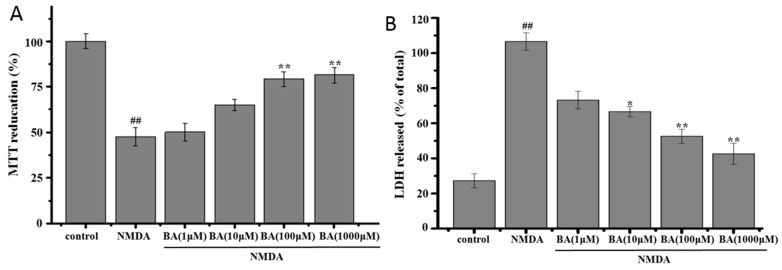

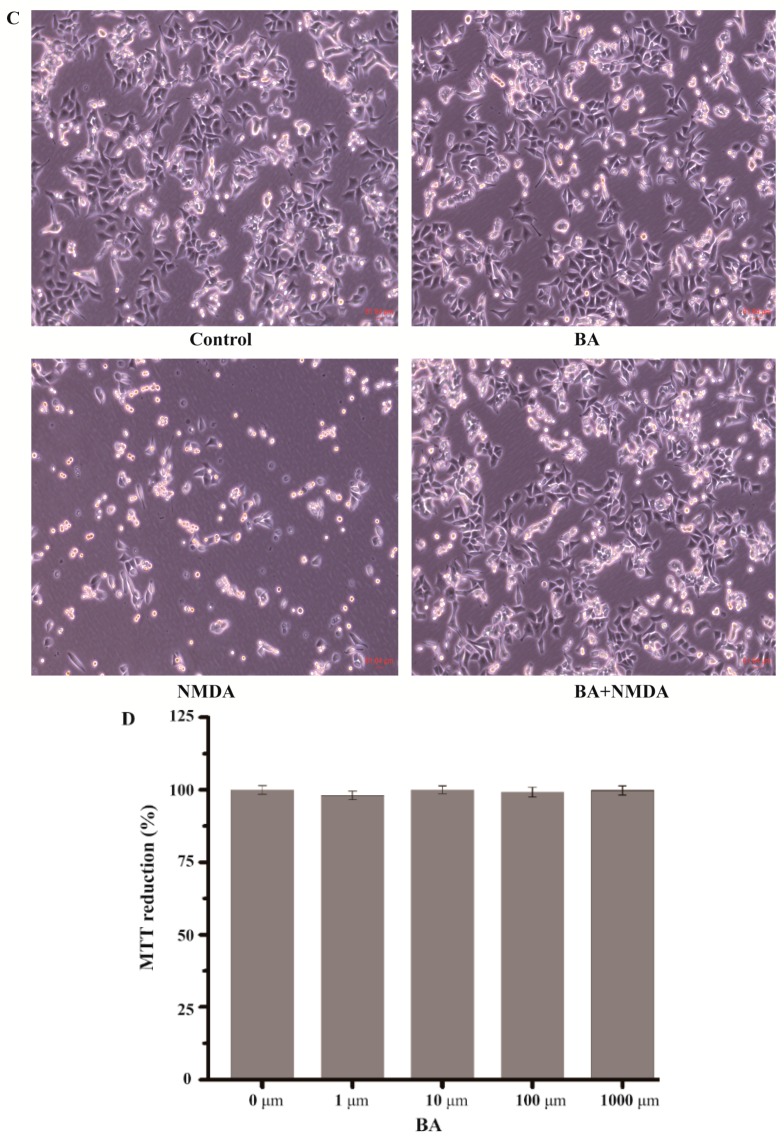
Effect of BA on NMDA-induced PC12 cells. After treatment of cells with different concentrations of BA for 24 h and 20 mM of NMDA for 6 h, cell viability was assessed via (**A**) MTT assay and (**B**) LDH assay, respectively; (**C**) The effects of BA in NMDA-induced cell morphological changes; (**D**) The effect of BA itself on basal growth in PC12. Data are represented as mean ± SD, *n* = 8 wells for each group. ^##^ *p* < 0.01 *vs.* control group. * *p* < 0.05, ** *p* < 0.01 *vs.* NMDA group.

The effect of BA on the cell viability in NMDA-induced excitotoxicity in PC12 cells is shown in Figure 3. Cell viability exposed to 20 mM NMDA for 6 h was 51.6% ± 2.8% of the control value, and cells shrank and changed greatly in morphology (Figure 3C), but cell viability was raised and the morphology was protected when cells were pretreated with different concentrations of BA prior to exposure to NMDA (Figure 3A,B). BA at 100 µM and 1000 µM could significantly inhibit NMDA-induced excitotoxicity. Additionally, the effect of BA itself on basal growth in PC12 was tested. Results indicate that BA did not have an inhibitional effect on basal growth in PC12 (Figure 3D).

### 2.2. Effect of BA on LDH Release

As shown in Figure 3B, treatment with NMDA (20 mM) resulted in an increase of LDH release, which was 106.5% ± 11.3% higher than control. Pre-incubation with BA at a different concentration obviously blocked LDH leakage compared to NMDA cells (*p* < 0.05 or *p* < 0.01). The results were similar to those determined by the MTT assay.

### 2.3. Effect of BA on Intracellular Ca^2+^ Concentration

**Figure 4:**
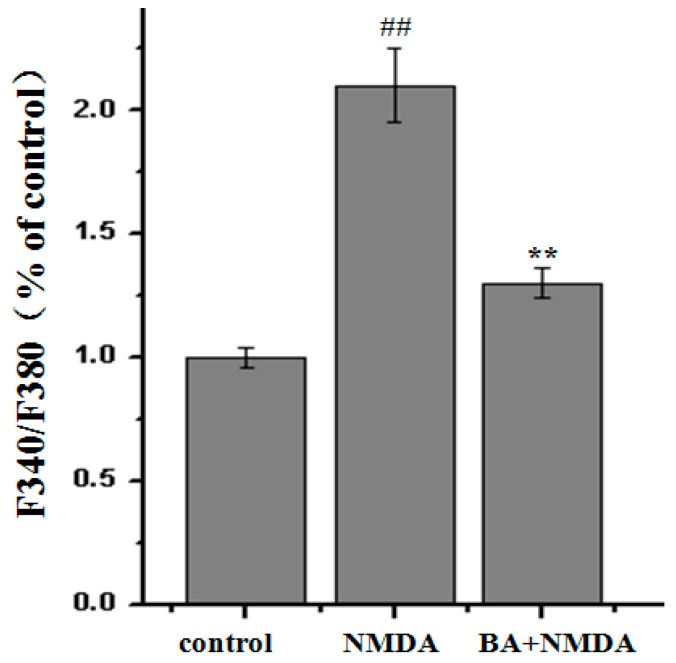
The effect of BA (100 μM) on intracellular Ca^2+^ concentration in NMDA-induced PC12 cells. Intracellular Ca^2+^ concentration was measured by the Fura-2/AM fluorescent technique. Values given are the mean ± SD (*n* = 6). ^##^ *p* < 0.01 *vs.* control group; ** *p* < 0.01 *vs.* NMDA group.

From Figure 4, we could see that the fluorescence intensity of Fluo-2AM increased after NMDA treatment compared with control group; however, BA (100 µM) pretreatment decreased the fluorescence intensity. The NMDA treatment group significantly increased the fluorescence intensity of intracellular Ca^2+^ from 263.8% ± 40.1% to 567.9% ± 28.6%. Additionally, pre-incubation with BA (100 µM) at the different concentration obviously decreased fluorescence intensity of intracellular Ca^2+^ relative to the control group (326.6% ± 51.5%).

### 2.4. Effects of BA on the Protein Expression of CaMKII, p-CaMKII, CREB, p-CREB, Bax, Bcl-2

**Figure 5:**
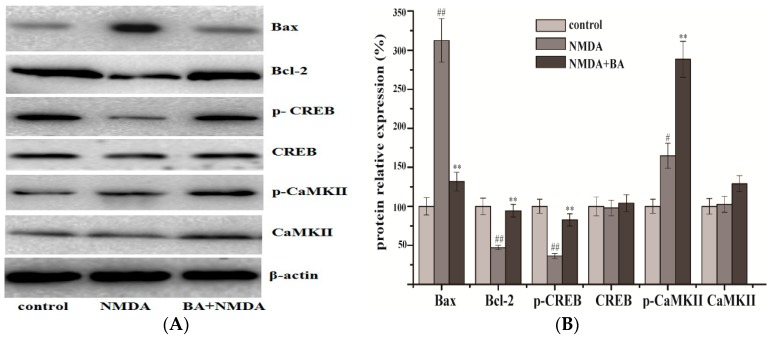
Effect of BA (100 μM) on protein levels in PC12 cells. (**A**) The level of CaMKII, p-CaMKII, CREB, p-CREB, Bax, and Bcl-2 was determined by Western blot. β-actin was used as the internal control; (**B**) The relative optical densities were indicated. Values given are the mean ± SD (*n* = 3). ^#^ *p* < 0.05, ^##^ *p* < 0.01 *vs.* control group; ** *p* < 0.01 *vs.* NMDA group.

As shown in Figure 5, levels of Bcl-2 and p-CREB protein expression obviously decreased in the model group compared with the control group, while the BA (100 µM) treatment groups had upregulated expressions of them. On the other hand, the level of Bax increased in the model group compared with the control group, but was downregulated in the BA (100 µM) treatment. Expression of CaMKII and CREB showed no significant change both in the model and in the treatment groups.

### 2.5. Effects of BA on the mRNA Expression of CaMKII, CREB, Bax, Bcl-2

**Figure 6:**
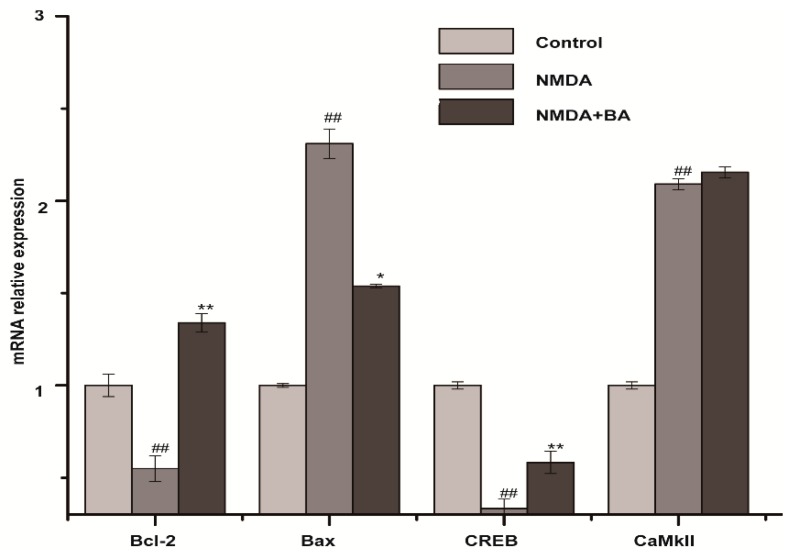
Effect of BA (100 μM) on mRNA levels in PC12 cells. The level of CaMKII, CREB, Bax, and Bcl-2 was determined by real-time PCR. GAPDH was used as the internal control. ^##^ *p* < 0.01 *vs.* control group; * *p* < 0.05, ** *p* < 0.01 *vs.* NMDA group.

As shown in Figure 6, levels of Bcl-2 and CREB mRNA expression decreased and that of Bax mRNA increased in the model group. While BA (100 µM) co-treatment suppressed the NMDA-induced increase in Bax and decrease in Bcl-2 and CREB, CaMKII was almost the same in the model and BA groups.

## 3. Discussion

To date, stroke/cerebral ischemia is the third leading cause of mortality and morbidity, behind cancer and heart disease. Due to little drugs currently available for the clinical treatment of acute stroke, it is of great interest. Exitotoxicity is considered as a crucial mechanism of neuronal death in cerebral ischemia [35]. NMDA is a kind of excitatory amino acid, which acts on *N*-methyl-d-aspartate receptors (NMDAR) of postsynaptic neuron, activates calcium channel controlled by receptor, and results in calcium overload. Much Ca^2+^ internal flow leads to acute edema in the cell, and secondary cell toxicity ultimately triggers neuron apoptosis.

PC12 cells, which are widely used in *in vitro* neural system disease research, are derived from *Rattus norvegicus* pheochromocytoma tumors. A high concentration of NMDA-induced excitotoxicity leading to neuronal damage in the PC12 cell line has been used as an *in vitro* experimental model of cerebral ischemia [36,37]. In our study, PC12 cell viability was significantly inhibited by NMDA in the NMDA treatment group relative to the control group. Thus, we believe that it is suitable to investigate whether BA has protective effects against NMDA-induced excitotoxicity in PC12 cells.

Triterpenoids are considered one of the most functionally and structurally diverse classes of secondary metabolites. They are usually cyclized from oxidosqualene and form diverse triterpene skeletons. Moreover, they possess a variety of biological activities, such as those that are anti-inflammatory, hepatoprotective, and analgesic, because of their impressive skeletal diversity [38]. BA (3-hydroxy-D:C-friedoolean-8-en-29-oic acid) is a triterpenoid and has unique chemical attributes within the triterpenoid family (namely, the unsaturated B−C ring fusion). It usually possesses an interesting pleiotropic profile of biological activity. Additionally, our previous experiment showed it improved the cell viability of PC12 cells. It is interesting and worth researching.

The experiment results showed that LDH release in the BA treatment group decreased, and cell viability increased. It was suggested that BA had a protective effect against NMDA. While PC12 cells are a cell line that has neuron phenotypes, it cannot be completely on behalf of primary cultured neurons. Therefore, further work needs to focus on an *in vivo* primary cultured neuron model directly.

As already stated, when intracellular Ca^2+^ concentration is 10^−6^ mol/L or above, CaM is activated by combining with Ca^2+^, Ca^2+^/CaM activates CaMKII, and further autophosphorylates at Thr286 to render the kinase constitutively active, then produces substantial Ca^2+^/calmodulin-independent activity. The present study showed that NMDA induced phosphorylation of the transcription factor CREB and CaMKII in PC12 and promoted Bax generation, but inhibited Bcl-2 expression in PC12. Although expression level of p-CREB, p-CaMKII, and Bcl-2 was increased by BA, Bax was upregulated. These results are consistent with previous research. They provide additional data and substantiate previous observations showing that radices trichosanthis could lighten apoptosis in rats after cerebral ischemia reperfusion. The results suggest that p-CREB mediated neuroprotection in the Ca^2+^-CaMKII-CREB signal pathway.

## 4. Materials and Methods

### 4.1. Materials

BA was extracted and separated by the College of Pharmacy of Fujian University of Traditional Chinese Medicine and was identified via ^13^C-NMR. Purity of all BA was more than 98% via HPLC analyses. Rat adrenal pheochromocytoma (PC12) cells were obtained from Beijing North Carolina Chuanglian Biotechnology Research Institute (Beijing, China). RPMI-1640, fetal bovine serum, penicillin, and streptomycin were obtained from HyClone (Logan, UT, USA). NMDA, 3-(4,5-dimethylthiazol-2-yl)-2,5-di-phenyl (MTT), and Fura-2/AM were purchased from Sigma Aldrich (St Louis, MO, USA). The lactate dehydrogenase (LDH) assay kit was bought from Jiancheng Institute of Biological Engineering (Nanjing, China). Antibodies to CaMKII, p-CaMKII, CREB, p-CREB, Bax, Bcl-2, and actin were bought from Cell Signaling Technology, Inc. (Boston, USA). The secondary antibodies were from Xiamen Lulong Biotech Co., Ltd. (Xiamen, China). Polyvinylidene fluoride membrane was from Merck KGaA (Darmstadt, Germany). TRIzol Reagentand cDNA reverse transcription kit was bought from Thermo Fisher Scientific Co., Ltd. (Beijing, China). All other reagents were from Beyotime Institute of Biotechnology (Nanjing, China) unless otherwise stated.

### 4.2. Cell Culture and Treatment

PC12 cells were cultured in RPMI 1640 medium supplemented with 10% (*v*/*v*) fetal bovine serum, 100 U/mL penicillin, and streptomycin at 37 °C in humidified atmosphere of 95% air and 5% CO_2_. After seeding onto 96-well plate or 6-well plate for 24 h, the cells were cultured in medium without serum and incubated with the presence or absence of different concentrations of BA for 24 h. Then, proper concentration NMDA was then added for an additional time.

### 4.3. Cell Viability Assay

Conventional MTT reduction assay was used to determine cell viability. PC12 cells were seeded in 96-well plates at a density of 2 × 10^4^ cells per well. After treatment, 10 µL MTT (0.5 mg/mL) was added to each culture well for an additional 4 h incubation. Then, the MTT was removed, and the cells were dissolved with 100 µL of dimethylsulfoxide. Absorbance at 570 nm was measured in a microplate reader (Infinite M200 Pro, Tecan, Switzerland) after the formazan dye crystals were solubilized. Cell viability was expressed as a percentage of non-treated control.

### 4.4. Lactate Dehydrogenase (LDH) Assay

LDH assay was employed to assess the cytotoxicity of drug. PC 12 cells were seeded in 96-well plates at a density of 2 × 10^4^ cells per well. After treatment , the medium was collected to measure the LDH activity according to the manufacturer’s protocol. Absorbance at 450 nm was measured in a microplate reader (Infinite M200 Pro). LDH leakage was expressed as the percentage (%) of the total LDH activity (LDH in the medium + LDH in the cell).

### 4.5. Measurement of Intracellular Ca^2+^ Concentration

Intracellular Ca^2+^ concentration was determined as described in previous literature [12]. PC12 cells were seeded in 96-well plates (flat bottom black, clear bottom polystyrol) at a density of 2 × 10^4^ cells per well. After treated with BA (100 µM) for 24 h in the presence or absence of NMDA for an additional 6 h, the cells were washed with D-PBS and incubated with the complete medium containing 5 µm Fura-2/AM at 37 °C for 45 min. Subsequently, the cells were washed with D-PBS containing 0.2% bovine serum albumin. Then, the cells were incubated at 37 °C for another 5 min prior to measurement. Intracellular Ca^2+^ concentration was determined by alternating excitation wavelengths of between 340 and 380 nm with emission at 510 nm in a fluorescence spectrophotometer (Infinite M200 Pro). Intracellular Ca^2+^ concentration was expressed as a percentage of non-treated control.

### 4.6. Western Blot Analysis

Western blot analysis was used to evaluate protein level. PC12 cells were seeded in 6-well plates at a density of 2 × 10^5^ cells per well. After treatment, cells were collected and lysed via lysis buffer, and they were then centrifuged at 12,000 *g* for 15 min. The supernatant was collected and the protein concentration was determined via the BCA method. Then, protein was mixed with loading buffer and incubated in 100 °C for 6 min. Finally, samples were stored at −20 °C for further analyses.

Equal amounts of protein (20 µg) were electrophoresed on 12% density SDS acrylamide gels. Following electrophoresis, the proteins were transferred from the gel to PVDF membranes using an electric transfer system. Non-specific binding was blocked with 5% skim milk in TBST buffer for 2 h. Then, PVDF membranes were incubated with antibodies to CaMKII (1:1000), p-CaMKII (1:1000), CREB (1:1000), p-CREB (1:1000), Bax (1:1000), Bcl-2 (1:1000), and actin (1:1000) overnight at 4 °C. After that, they were washed 3 times with TBST, 10 min each time. Then, PVDF membranes were incubated for 2 h at room temperature with a second antibody (1:7000), after which they were washed 3 times with TBST, 10 min each time. Finally, they were evaluated using the ECL Western detection reagents. Three repeats of the experiments were performed.

### 4.7. Real-Time PCR Analysis

Real-time PCR analysis was used to evaluate mRNA level. PC12 cells were seeded in 6-well plates at a density of 2 × 10^5^ cells per well. After treated, cells were collected and total RNA was isolated from the cells with TRizol Reagent. Two micrograms of total RNA was reverse-transcribed to cDNA according to the manufacturer’s protocol. cDNA was subjected to real-time PCR assays with Power SYBR^®^ Green PCR Master Mix (Thermo Fisher Scientific (China)Inc., Shanghai, China). The specific sequences of primers are as follows: CREB, 5’-TAC CCA GGG AGG AGC AAT AC-3’ (forward) and 5’-GAG GCA GCT TGA ACA ACA AC-3’ (reverse); CaMKIIα, 5’-GAA GCC ATA AGC AAT GGA GA-3’ (forward) and 5’-GGT TTT CAA AAT AGA ATC GAT G-3’ (reverse); BCL-2: 5’-GGT GGT GGA GGA ACT CTT CA-3’ (forward) and 5’-ATG CCG GTT CAG GTA CTC AG-3’ (reverse); Bax: 5’-TGC AGA GGA TGA TTG CTG AC-3’ (forward) and 5’- GAT CAG CTC GGG CAC TTT AG-3’ (reverse); GAPDH, 5’-AGC CCA GAA CAT CAT CCC TG-3’ (forward) and 5’-CAC CAC CTT CTT GAT GTC ATC-3’ (reverse). Real-time quantitative PCR analysis was performed by using the ABI 7900 Real-Time PCR System (Applied Biosystems, Inc., Foster City, CA, USA) with the following procedure: 2 min hold at 50 °C, 10 min hold at 95 °C, followed by 60 cycles of 15 s at 95 °C and 1 min at 60 °C. The expression levels mRNA were recorded and normalized with the internal control (GAPDH).

### 4.8. Statistical Analysis

Data were expressed as mean ± SD. One-way analysis of variance (ANOVA) (SPSS16.0 statistical software, Chicago, IL, USA) followed by a *post hoc* LSD test to evaluate multiple group difference. Difference was considered statistically significant when *p* < 0.05.

## 5. Conclusions

In conclusion, BA has a protective effect on NMDA-induced excitotoxicity in PC12 cells. It was mediated by Ca^2+^ influx and gene expression on the Ca^2+^-CaMKII-CREB signal pathway. BA may be a potential neuroprotective compound for protection apoptosis, and Ca^2+^-CaMKII-CREB signal pathway may be one of the action pathways accounting for the protection against the *in vivo* excitotoxicity of BA.

## Abbreviations

The following abbreviations are used in this manuscript:

CaMKII: Ca^2+^/calmodulin-dependent protein kinase II
CREB: cAMP response element-binding protein
NMDA: *N*-Methyl-d-aspartate
PC12: pheochromocytoma cell line
BA: bryonolic acid
MTT: methyl thiazolyl tetrazolium
